# In_2_S_3_@TiO_2_/In_2_S_3_ Z-Scheme Heterojunction with Synergistic Effect for Enhanced Photocathodic Protection of Steel

**DOI:** 10.3390/molecules28186554

**Published:** 2023-09-10

**Authors:** Yue Chang, Kaili Suo, Yuhang Wang, Xiaona Ren, Jiangli Cao

**Affiliations:** 1 Institute for Advanced Materials and Technology, University of Science and Technology Beijing, Beijing 100083, China; 2 National Materials Corrosion and Protection Data Center, University of Science and Technology Beijing, Beijing 100083, China; 3 BRI Southeast Asia Network for Corrosion and Protection (MOE), Shunde Innovation School, University of Science and Technology Beijing, Foshan 528399, China; 4 Institute of Powder Metallurgy and Advanced Ceramics, School of Materials and Engineering, University of Science and Technology Beijing, Beijing 100083, China

**Keywords:** photocathodic protection, Z-scheme, In_2_S_3_, TiO_2_, 304 stainless steel

## Abstract

In this work, a TiO_2_/In_2_S_3_ heterojunction film was successfully synthesized using a one-step hydrothermal method and applied in the photocathodic protection (PCP) of 304SS. The octahedral In_2_S_3_ and In_2_S_3_@TiO_2_ nanoparticles combined and coexisted with each other, with In_2_S_3_ quantum dots growing on the surface of TiO_2_ to form In_2_S_3_@TiO_2_ with a wrapping structure. The composite photoelectrode, which includes TiO_2_ with a mixed crystalline phase and In_2_S_3_, exhibited significantly enhanced PCP performance for 304SS compared with pure In_2_S_3_ and TiO_2_. The In_2_S_3_@TiO_2_/In_2_S_3_ composites with 0.3 g of P25 titanium dioxide (P25) showed the best protection performance, resulting in a cathodic shift of its OCP coupled with 304SS to −0.664 V_AgCl_. The electron transfer tracking results demonstrate that In_2_S_3_@TiO_2_/In_2_S_3_ forms a Z-scheme heterojunction structure. The enhanced PCP performance could be attributed to the synergistic effect of the mixed crystalline phase and the Z-scheme heterojunction system. The mixed crystalline phase of TiO_2_ provides more electrons, and these electrons are gathered at higher energy potentials in the Z-scheme system. Additionally, the built-in electric field further promotes the more effective electrons transfer from photoelectrode to the protected metals, thus, leading to enhanced photoelectrochemical cathodic protection of 304SS.

## 1. Introduction

Corrosion of metals is common in nature because of a thermodynamically favorable spontaneous reaction. The harsh marine environment can accelerate the metal ions’ dissolution, and promote the development of corrosion. Environmental pollution, engineering safety accidents, and economic losses caused by corrosion have caused great harm to human activities. Photoelectrochemical cathodic protection is considered as one of the most promising and environmentally friendly strategies for protecting metals in service, especially in marine environments with sufficient sunlight. This is due to the non-sacrificial anode and the utilization of solar energy [1,2]. In this technology, the photogenerated electrons produced by semiconductor photoanodes are transferred to the coupled metal to prevent corrosion, helping it achieve a thermodynamically stable state. The photoelectric characteristics of semiconductors directly determine the photoelectrochemical cathodic protection effect on metals, and the semiconductors (or photoelectrodes) play a crucial role in the entire protection process [3,4].

Since the report of photoelectrochemical cathodic protection of copper using TiO_2_ by Yuan and Tsujikawa in 1995 [5], titanium dioxide (TiO_2_), one of the most important n-type semiconductors, has been widely investigated due to its efficient photocatalytic properties, good stability, low toxicity, and cost-effectiveness. A series of TiO_2_-based photoanodes have been fabricated and applied in photocathodic protection for metals [6,7,8,9]. However, because of low light-harvesting efficiency, the high recombination efficiency of photogenerated carriers, and an insufficiently negative Fermi level, developing a highly efficient semiconductor photoanode for photoelectrochemical cathodic protection on metals remains a significant challenge [10,11]. 

Many efforts have been made to modify TiO_2_ [12], such as morphology control, elemental doping, and heterojunction construction, to improve the performance of photoelectrochemical cathodic protection [13,14,15,16]. Among these methods, the combination of TiO_2_ with narrow-band-gap semiconductors to form a heterostructure has shown to be an effective approach for enhancing light absorption and carrier separation efficiency. In this case, indium sulfide (In_2_S_3_) stands out as an ideal candidate due to its narrow band gap (2.1~2.2 eV) and a Fermi level that is more negative than that of TiO_2_ [17,18,19]. Huang et al. reported the synthesis of a type II In_2_S_3_/TiO_2_ heterostructure to enhance activity in CO_2_ photocatalytic reduction [20]. Park et al. prepared a nanostructured type II In_2_S_3_/TiO_2_ photoanode using the hydrothermal method for efficient photoelectrochemical water splitting [21]. While type II In_2_S_3_/TiO_2_ materials have exhibited good photocatalytic performance in CO_2_ reduction and water oxidation, they are not ideal for the photocathodic protection of 304 stainless steel (304SS) [22]. In addition to type II photocatalysts, the Z-scheme photocatalyst system is another heterojunction structure that consists of two semiconductors mimicking the natural photosynthesis of green plants. The electrons in the higher conduction band and the holes in the lower valence band would participate in photocathodic protection [23,24,25]. Z-scheme heterostructures can achieve more efficient charge separation and more negative reduction potential for photogenerated electrons, demonstrating their evident superiority compared with type II photocatalysts. However, there are few reports on Z-scheme In_2_S_3_/TiO_2_ materials for photocathodic protection. Furthermore, P25 TiO_2_, a commonly used commercial photocatalyst, is known to exhibit more excellent photocatalytic performance than anatase TiO_2_ or rutile TiO_2_ due to its mixed crystalline phase effect. However, the underlying influence of the mixed crystalline phase in P25 TiO_2_ on charge transfer in Z-scheme systems remains unclear.

Inspired by the above discussion, in this work, we report a direct Z-scheme In_2_S_3_@TiO_2_/In_2_S_3_ system to enhance the photoelectrochemical cathodic protection performance and protect 304SS. The In_2_S_3_@TiO_2_/In_2_S_3_ composite was successfully prepared on the fluorine-doped tin oxide (FTO) glass substrates through a simple one-step hydrothermal method. We investigated the morphologies, crystalline structures, and optical properties of the resulting In_2_S_3_@TiO_2_/In_2_S_3_ composites. The influence of TiO_2_/In_2_S_3_ heterojunction and crystalline phase of TiO_2_ on the PCP performance of the as-prepared composite films for 304SS was also examined. Furthermore, the charge transfer mechanism was explored to clarify the promoting mechanism of In_2_S_3_@TiO_2_/In_2_S_3_ in photocathodic protection of 304SS. 

## 2. Results and Discussion

### 2.1. Characterization of the As-Synthesized Samples

**Figure 1:**
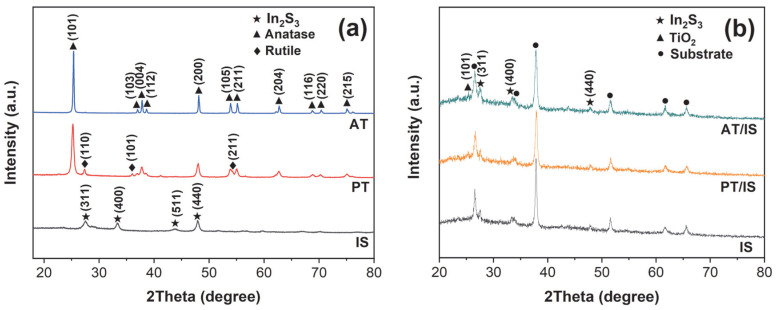
(**a**) The XRD spectra of anatase TiO_2_ (AT), P25 TiO_2_ (PT), and In_2_S_3_ (IS) powder samples; (**b**) the XRD spectra of AT/IS, PT/IS, and IS samples on FTO substrates.

The crystal structures of the purchased AT and PT and the hydrothermally obtained IS powders were characterized by XRD and the results are shown in Figure 1a. It is evident that all the observed diffraction peaks of AT samples correspond to the anatase phase of TiO_2_ (JCPDS, No. 21-1272) [26]. In addition to the diffraction peaks of the anatase phase, the diffraction peaks at 27.4°, 36.1°, and 54.3° in the XRD pattern of PT samples belong to (110), (101), and (211) lattice planes of the rutile phase (JCPDS, No. 21-1276) [19]. As for the hydrothermally obtained IS samples, the diffraction peaks at 27.4°, 33.2°, 43.6°, and 47.7° can be attributed to the β-In_2_S_3_ (JCPDS, No. 65-0459) crystal planes of (311), (400), (511), and (440) [27], indicating the successful synthesis of In_2_S_3_ samples. The XRD patterns of the prepared AT/IS, PT/IS, and IS coatings are shown in Figure 1b. It is worth noting that all the spectra for the three kinds of coatings display diffraction peaks corresponding to the FTO substrates (marked with ●). After deducting the FTO substrate, the diffraction peaks of the IS coatings can be indexed to the characteristic peaks of β-In_2_S_3_. For the AT/IS and PT/IS coatings, the diffraction peaks ascribed to both β-In_2_S_3_ and anatase phases are identified. However, there are no detectable peaks of the rutile phase for PT/IS samples, which could be attributed to the low peak intensity of the rutile phase in PT/IS samples.

**Figure 2:**
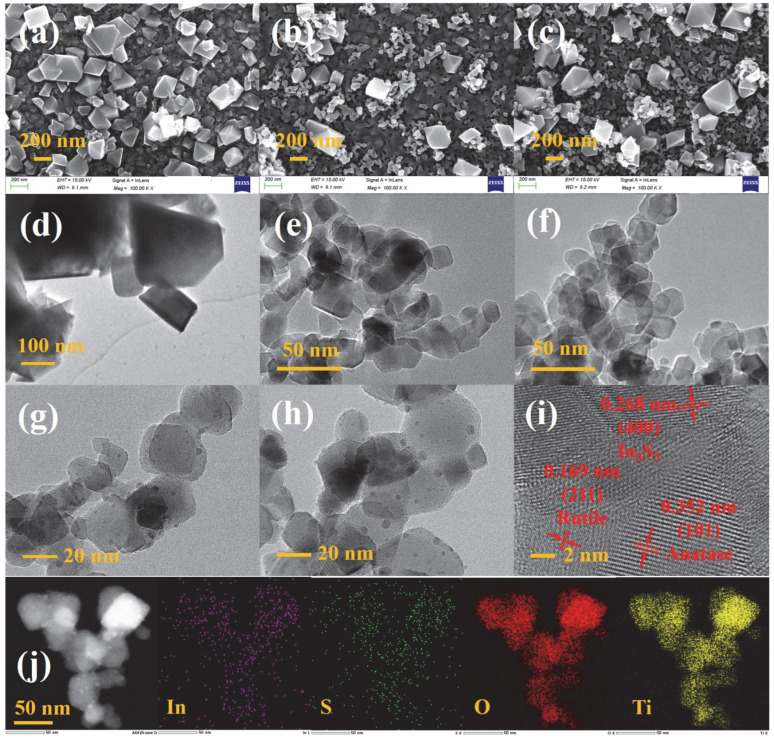
SEM images of (**a**) IS, (**b**) AT/IS, and (**c**) PT/IS samples on FTO substrates; TEM images of (**d**) IS, (**e**) AT, (**f**) PT, (**g**) AT/IS, and (**h**) PT/IS samples; (**i**) HRTEM image and (**j**) EDS mapping images of PT/IS samples.

The morphologies of IS, AT/IS, and PT/IS on FTO substrates were observed by SEM, and the obtained images are shown in Figure 2a–c. The hydrothermally synthesized In_2_S_3_ samples show octahedron or truncated octahedron shapes, with particle sizes ranging from 200 to 500 nm (Figure 2a). As shown in Figure 2b,c, the AT/IS and PT/IS composites have a similar morphology, with the nanoparticles of TiO_2_ dispersed on the surface and around these octahedral materials. Table S1 shows the AT/IS and PT/IS film roughness obtained by the analysis of AFM characterization results (Figure S1). The R_q_ of AT/IS and PT/IS films are about 120~130 nm. The TEM images of pure In_2_S_3_ and TiO_2_ shown in Figure 2d–f, and the edges and corners of In_2_S_3_ are clearly seen in Figure 2d, consistent with the results obtained from the SEM image in Figure 2a. The particle sizes of AT and PT samples are both 20~30 nm in Figure 2e,f. Observing the images of TEM for AT/IS and PT/IS composites in Figure 2g,h, the quantum dots with the particle sizes of 2~6 nm appear on the surface of TiO_2_. Furthermore, as shown in Figure 2i, there are three different lattice fringes that can be found in the HRTEM of PT/IS samples. The lattice fringe spacings of 0.169 nm and 0.352 nm correspond to the (211) plane of rutile TiO_2_ and the (101) plane of anatase TiO_2_ [20], while the lattice fringe spacing of 0.268 nm corresponds to the (400) plane of In_2_S_3_ [25]. These results indicate that the quantum dots are In_2_S_3_. Additionally, TEM–EDS elemental mapping images of PT/IS composites, shown in Figure 2j, display the distribution of In, S, O, and Ti elements, with no other impurity elements detected. The quantum dots are uniformly dispersed on the surface of TiO_2_, forming an In_2_S_3_@TiO_2_ heterostructure. Therefore, these results evidently confirm the formation of the In_2_S_3_@TiO_2_/In_2_S_3_ heterojunction, indicating the successful preparation of the composite.

**Figure 3:**
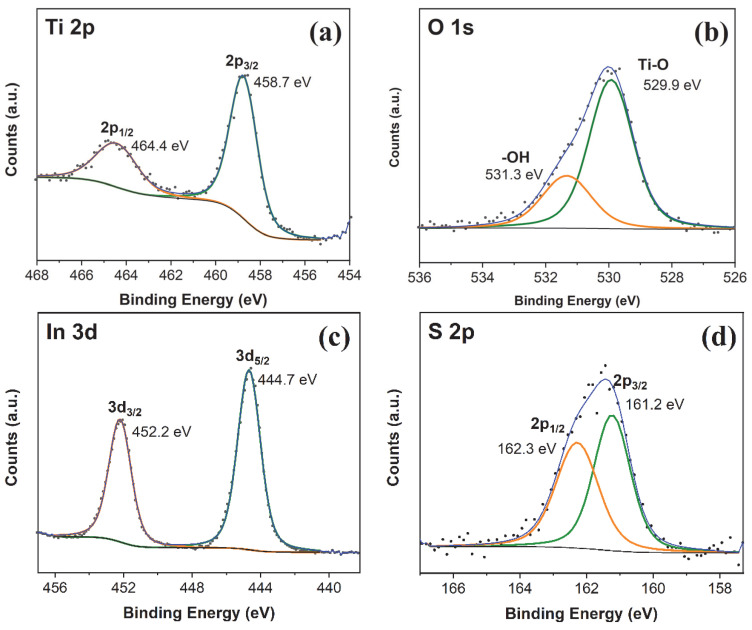
High-resolution XPS spectra of (**a**) Ti 2p, (**b**) O 1s, (**c**) In 3d, and (**d**) S 2p of PT/IS samples.

In order to investigate the surface elemental composition and chemical state of PT/IS, the XPS analysis was performed. Figure 3 shows the high-resolution XPS spectra of Ti 2p, O1s, In 3d, and S 2p. It can be seen from the Ti 2p XPS spectrum (Figure 3a), two peaks at 458.7 eV and 464.4 eV are observed, corresponding to Ti 2p_3/2_ and Ti 2p_1/2_, respectively, which can be attributed to Ti^4+^ of TiO_2_ [11,12,13,14,15,16,17,18,19]. The peaks in the O1s spectrum for PT/IS samples (Figure 3b) can be fitted into two peaks with binding energies centered at 529.9 eV and 531.3 eV. These peaks correspond to lattice oxygen species in TiO_2_ and surface hydroxyl groups/absorbed oxygen, respectively [28]. The In 3d XPS spectrum is shown in Figure 3c, with the peaks at 444.7 eV and 452.2 eV corresponding to the In^3+^ 3d_5/2_ and In^3+^ 3d_3/2_ peaks, respectively, indicating the presence of In^3+^ in PT/IS samples [17]. Figure 3d shows the S 2p XPS spectrum of PT/IS. After further fitting of the S 2p, two peaks of S 2p_3/2_ and S 2p_1/2_ appear at 161.2 and 162.3 eV, respectively, which is ascribed to S^2+^ of In_2_S_3_ [20]. The XPS results further confirm the successful synthesis of a composite consisting of TiO_2_ and In_2_S_3_. 

**Figure 4:**
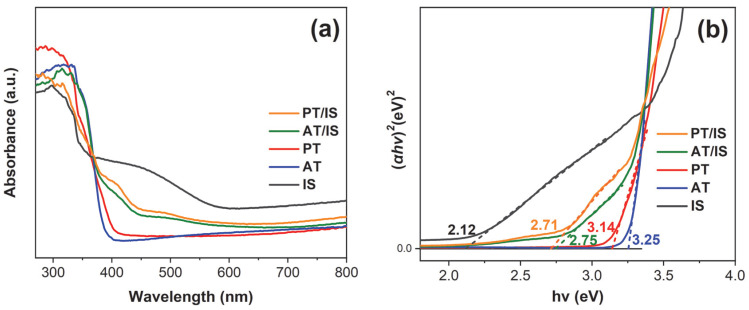
(**a**) UV–vis DRS spectra and (**b**) corresponding Tauc plots of IS, AT, PT, AT/IS, and PT/IS samples.

The UV–vis DRS spectra of IS, AT, PT, AT/IS, and PT/IS samples are shown in Figure 4a. It is evident that the optical absorption edge for AT and PT is located around 382 nm and 395 nm, respectively. The IS samples exhibit absorption in the visible light region, characterized by an absorption edge at ~585 nm. Upon the incorporation of In_2_S_3_, both AT/IS and PT/IS present a noticeable enhancement in response in the range of 400~600 nm compared with AT and PT, respectively. This indicates an improved light absorption capability of the AT/IS and PT/IS samples within the visible light region. Notably, the absorption edge of PT/IS has an obvious redshift compared with that of AT/IS, which can be attributed to the mixed crystalline phase of P25. Furthermore, as shown in Figure 4b, the band gap energies of the above samples were calculated based on the (α*hv*)^2^-*hv* curves [29]. The *E*_g_ values of AT and PT are ca. 3.25 eV and 3.14 eV, respectively, while that of IS is ca. 2.12 eV. This indicates that Ti elements are successfully doped into the bulk of α-Fe_2_O_3_, which slightly increases the visible light absorption.

### 2.2. The Influence of Heterojunction Structure on PT/IS with Mixed Crystalline Phase Photoelectrode

**Figure 5:**
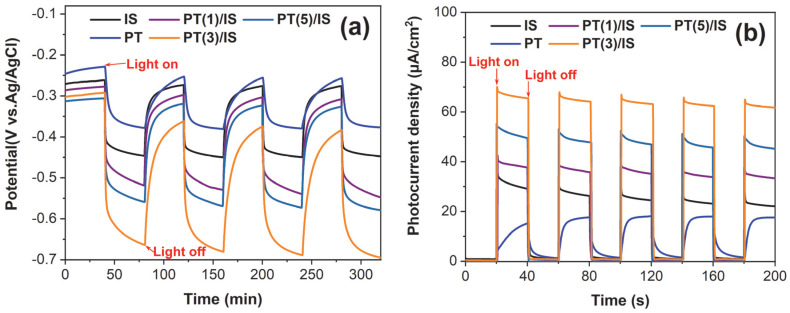

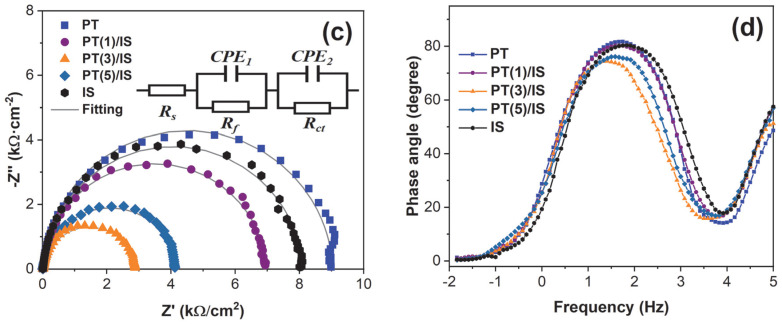
Photoinduced OCP–time curves (**a**), photocurrent density–time curves (**b**) of the prepared photoelectrodes including pure PT, pure IS, and PT/IS with different amounts of p25 added coupled with 304SS; Nyquist plots (**c**) and Bode phase angle plots (**d**) of the prepared photoelectrodes including pure PT, pure IS, and PT/IS with different amounts of p25 added under light irradiation.

A series of PT/IS photoelectrodes with different amounts of added P25 (0.01 g, 0.03 g, 0.05 g) were prepared and designated as PT(1)/IS, PT(3)/IS and PT(5)/IS, respectively. The trends of OCP change for all the PT/IS photoelectrodes are shown in Figure 5a. All the photoelectrodes show a strong sensitivity to light irradiation, with a dramatic decrease in OCP from off illumination to on illumination. This decrease is attributed to the migration of photogenerated electrons from the photoelectrode to 304SS. When exposed to light, as the amount of P25 added increases from 0 to 0.03 g, the negative shift in the photo-potential for PT/IS photoelectrodes coupled with 304SS becomes more pronounced. This further confirms the positive effect of the heterojunction structure in PT/IS with a mixed crystalline phase, which promotes the fast injection of electrons from PT/IS to 304SS and subsequent cathodic polarization. However, when the amount of P25 added further increases, the negative shift in the photo-potential decreases. This could be attributed to the excessive P25 addition, leading to the aggregation of nanoparticles and possibly becoming recombination centers for electron–hole pairs. In addition, the photocurrent densities of different PT/IS photoelectrodes coupled with 304SS under intermittent solar light are shown in Figure 5b. As observed, all the samples generate positive photocurrents, indicating the migration direction of photogenerated electrons from the photoelectrodes to 304SS. The PT(3)/IS photoelectrode presents the highest photocurrent response, consistent with the results of the above OCP measurements, further emphasizing the important role of the heterojunction in the PT/IS system.

To gain a deeper understanding of the PT/IS heterojunction, the electron transport behavior and electron lifetime were investigated though EIS measurements under light irradiation at an open-circuit voltage. The Nyquist and corresponding Bode phase angle plots of pure PT, IS, and composite PT/IS photoelectrodes are shown in Figure 5c,d. An equivalent circuit model with a two-time constants for EIS data fitting is exhibited in the inset of Figure 5c and the values of fitting circuit parameters are listed in Table 1. R_s_, R_f_, R_ct,_ and CPE represent the series resistance, the film resistance, the charge transfer resistance across the interface of the photoanode and electrolyte, and the constant phase element, respectively [8,9,10,11,12,13,14,15,16,17,18,19,20,21,22,23,24,25,26,27,28,29,30]. The charge transfer resistance across the interface of the photoelectrode and electrolyte in the low-frequency region is an important parameter for evaluating the electrochemical behavior of photoelectrodes. The composite PT/IS reduces R_ct_ compared to pure PT and IS, indicating a faster charge transfer in the composite PT/IS owing to the formation of a heterojunction structure between TiO_2_ with a mixed crystalline phase and In_2_S_3_. The R_ct_ value for PT(3)/IS composite is 2920 Ω, which is lower than that of the other PT/IS composites. This result is consistent with the findings of the PEC performance tests, including OCP and photocurrent response test. 

Moreover, according to the Bode phase angle plots of the photoelectrode samples under illumination, the lifetime of photogenerated electrons (*τ*) can be obtained through the following Equation (1):

(1) $$\tau =\frac{1}{2{\pi f}_{max}}$$

where $f_{max}$ represents the characteristic peak frequency at low frequency, which is the frequency value (Hz) corresponding to the first peak [31,32]. As shown in Figure 5d, the values of $f_{max}$ for PT(3)/IS composite photoelectrodes are smaller than those of pure photoelectrodes and the other PT/IS composite photoelectrodes. This indicates that the lifetime of the photogenerated electrons of PT(3)/IS composite is the longest, facilitating the efficient separation and transfer of photogenerated carriers. 

Additionally, the linear sweep voltammetry (LSV) curves of PT, IS, and PT(3)/IS photoelectrodes in 0.15 M Na_2_S + 0.1 M Na_2_SO_3_ solution are exhibited in Figure S2a. As a hole scavenger, Na_2_S and Na_2_SO_3_ can effectively trap the photogenerated holes that arrive at the surface. According to the previous reports [6,7,8,9,10,11,12,13,14,15,16,17,18], the charge separation efficiency can be calculated by the equation: ($\eta_{sep}=\frac{J^{HS}}{J_{abs}}$), and the results are exhibited in Figure S2b. *J_abs_* represents the photocurrent density converted from the absorbed photons, and *J^HS^* is the photocurrent densities measured in 0.15 M Na_2_S + 0.1 M Na_2_SO_3_ solution. PT(3)/IS presents a remarkably higher charge separation efficiency than PT and IS. This indicates that the heterojunction structure in the PT/IS system contributes to the effective separation of photogenerated charge.

Based on the above results, an adding amount of 0.03 g of TiO_2_ was used in the PT/IS and AT/IS samples in this work.

### 2.3. The Photocathodic Protection (PCP) Performance of PT/IS

**Figure 6:**
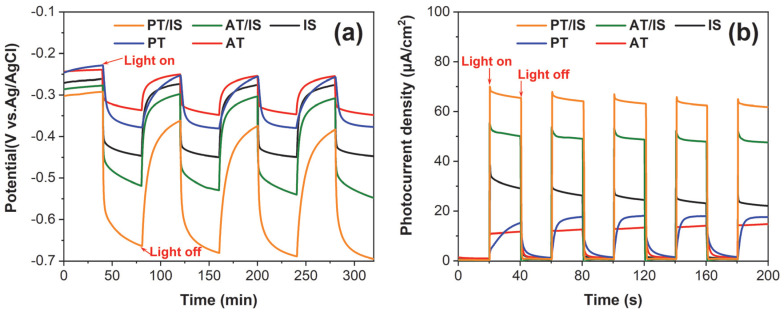
Photoinduced OCP–time curves (**a**), photocurrent density–time curves (**b**) of the prepared photoelectrodes including pure AT, PT, and IS, as well as composite AT/IS, PT/IS coupled with 304SS.

The photocathodic protection performance of the PT/IS samples was evaluated by measuring the photoinduced open-circuit potential (OCP) curves. For comparison, the pristine AT, PT, IS, and composite AT/IS were also measured under the same conditions. The time-dependent photoinduced OCP of the five photoelectrodes coupled with the 304SS is presented in Figure 6a. Under illumination, the potentials of IS, AT, PT, AT/IS, and PT/IS are −336 mV, –379 mV, −519 mV, and −664 mV, respectively. Meanwhile, the corresponding potential drops are 106 mV, 139 mV, 242 mV, and 372 mV, respectively. It can be observed that the composite photoelectrodes exhibit a more negative shift in potential compared with the pure photoelectrodes. This indicates that the formation of heterojunction structure of TiO_2_ and In_2_S_3_ promotes the effective separation and transfer of photogenerated electrons and holes. Furthermore, P25 TiO_2_-based photoelectrodes show higher PCP performance than those of anatase, as evidenced by the results of potential drops. This can be attributed to the mixed crystalline phase of TiO_2_, which enhances light absorption efficiency and allows the photoelectrode to provide more electrons for transport to 304SS. Notably, the difference in potential drop between PT/IS and PT is more pronounced than that between AT/IS and AT. This is mainly due to the synergistic effect of the mixed crystalline phase of rutile–anatase and the heterojunction structure of TiO_2_ and In_2_S_3_. In the TiO_2_/In_2_S_3_ system, the impact of the mixed crystalline phase on the electrons transport to 304SS may be amplified by the heterojunction structure. It is likely that the mixed crystalline phase provides catalytic hotspots and promotes efficient transfer of photogenerated electrons at the interface of rutile and anatase [33,34]. Moreover, the above study demonstrates that the heterojunction structure of TiO_2_/In_2_S_3_ can further accelerate electron transport to 304SS. Therefore, the PT/IS photoelectrode exhibits the best PCP performance.

Figure 6b displays the photocurrent densities of different photoelectrodes coupled with 304SS under intermittent solar light. The order of photocurrent densities for all the photoelectrodes is as follows: AT < PT < AT/IS < PT/IS. The composite photoelectrodes present higher photocurrent densities compared to pure TiO_2_ photoelectrodes, with the PT/IS photoelectrode achieving the highest photocurrent density. It suggests that more photogenerated electrons for PT/IS photoelectrode are transferred to 304SS because of the synergistic effect of the mixed crystalline phase and heterojunction structure. This results in a more significant negative shift in potentials for 304SS, further confirming the results of OCP.

### 2.4. The Charge Transfer Mechanism of PT/IS Photoelectrode

**Figure 7:**
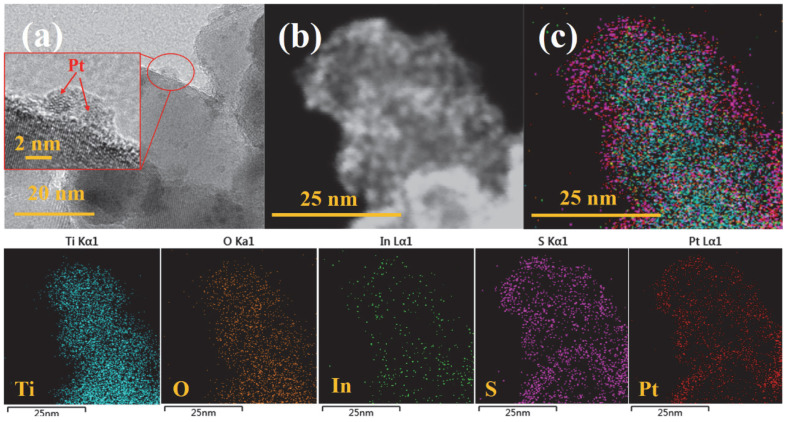
TEM image (**a**), electron image (**b**), and EDS layered and element mapping images (**c**) of Pt decorated PT/IS samples.

To study the synergistic effect of the mixed crystalline phase and heterojunction structure, the transfer process of photogenerated charge carriers was investigated in PT/IS composites under light irradiation. Pt nanoparticles decorated the PT/IS samples using the photo-deposition method. According to the previous reports [35,36], Pt nanoparticles could be selectively deposited at sites where photogenerated electrons flow. Thus, the loading position of Pt nanoparticles can track the transfer direction of photoexcited electrons. As seen in Figure 7, in the TiO_2_/In_2_S_3_ heterojunction structure, Pt nanoparticles were observed to grow on In_2_S_3_ and were deposited at electron-rich sites through a photoreduction reaction (Pt^4+^ + 4e →Pt^0^). The results of TEM and EDS element mapping images support the charge transfer tracking results, indicating that the photogenerated electrons tend to move from TiO_2_ to In_2_S_3_. In other words, TiO_2_ and In_2_S_3_ form a Z-scheme heterojunction in this case.

**Figure 8:**
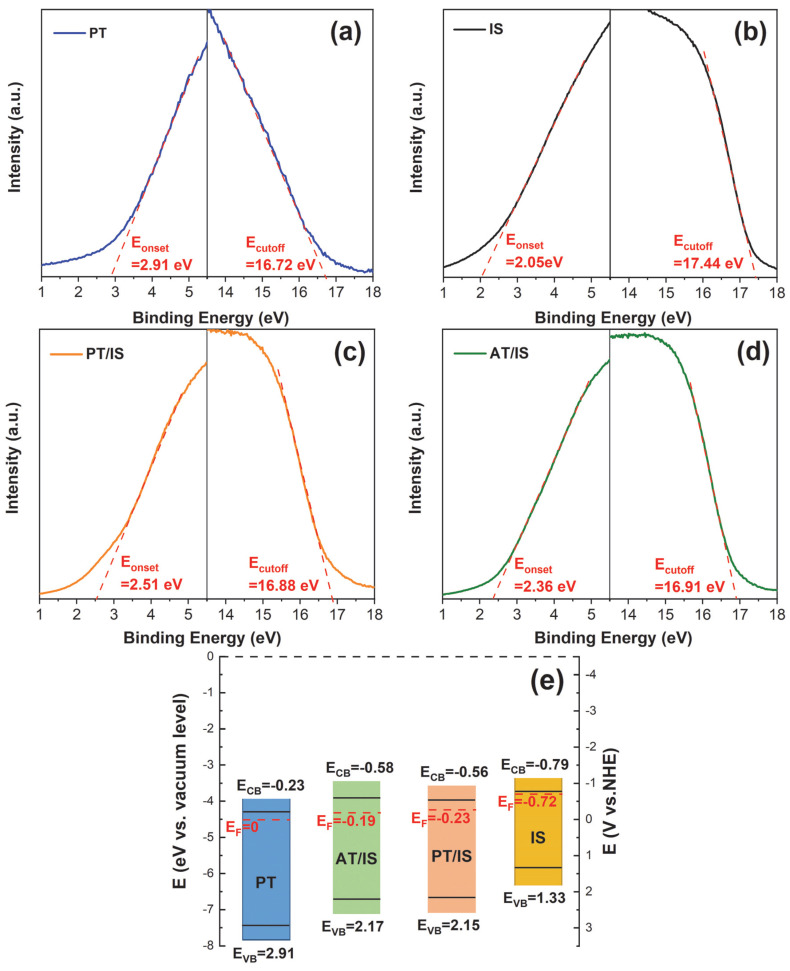
UPS spectra of pure (**a**) PT, (**b**) IS, and composite (**c**) PT/IS, (**d**) AT/IS samples; (**e**) band structures of PT, IS, AT/PT, and PT/IS.

To further elucidate the charge transfer mechanism, UPS spectra of AT, PT, IS, AT/IS, and PT/IS are presented in Figure 8a–d in order to obtain the Fermi level ($E_{F}$), the conduction band position ($E_{CB}$), and valence band position ($E_{VB}$). $E_{VB}$, $E_{CB}$, and $E_{F}$ were calculated according to Equations (2)–(5).

(2) $$E_{VB}=\left(E_{cutoff}-E_{onset}\right)-21.22$$

(3) $$E_{CB}=E_{VB}+E_{g}$$

(4) $$E_{F}=E_{VB}+E_{onset}$$

(5) $$E\left(vs.NHE\right)=-4.5\;eV-E(vs.\;vacuum\;level)$$

in which $E_{cutoff}$ and $E_{onset}$ refer to the cutoff binding energy and the onset binding energy, respectively [37,38]. As shown in Figure 8a–d, the *E_cutoff_* and *E_onset_* values of PT, IS, PT/IS, and AT/IS were given. According to Equations (2)–(5), the *E_VB_*, *E_CB_*, and *E_F_* of PT, IS, PT/IS, and AT/IS were calculated and the results are illustrated in Figure 8e. The Fermi levels of PT and IS are 0 V and −0.72 V, respectively. When PT and IS come into contact to form a heterojunction structure, the difference in Fermi levels between PT and IS leads to an interfacial electron transfer to maintain charge equilibrium. In this case, the *E_F_* value of the PT/IS sample becomes −0.23 V. It is noteworthy that this *E_F_* value is more negative than that of AT/IS (−0.19 eV) due to the mixed crystalline phase effect of PT, which facilitates the migration of photogenerated electrons from PT/IS to 304SS.

**Figure 9:**
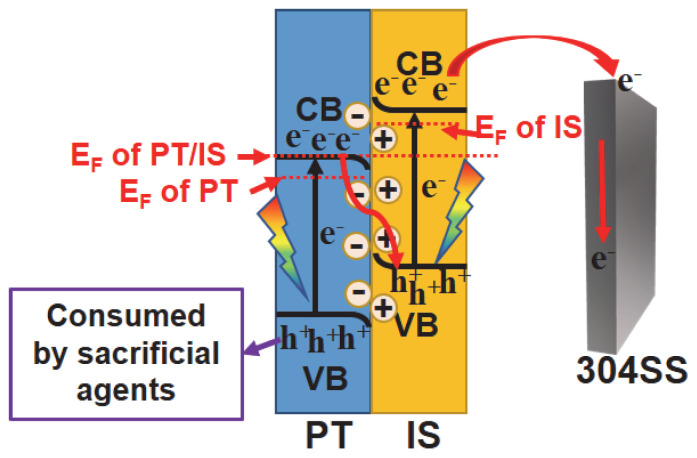
The enhanced PEC mechanism of PT/IS coupled with 304SS under light irradiation.

Based on the discussions above, Figure 9 illustrates a possible charge transfer process in the In_2_S_3_/TiO_2_ composite and the corresponding energy band structure of semiconductors to clarify the mechanism behind the enhanced performance of PT/IS for photocathodic protection of 304SS. The valence band (VB) edge potentials of PT and IS were obtained from UPS spectra, while the conduction band (CB) edge potentials of these semiconductors were calculated using the band-gap data obtained from UV–vis DRS characterization. The difference in electronic structure results in band bending of PT and IS, creating a built-in electric field at the heterojunction interface. As shown in Figure 9, under light irradiation, PT and IS become excited, generating electron–hole pairs in the CB and VB of the semiconductors, respectively. Due to the Z-scheme heterojunction and the built-in electric field, photogenerated electrons from PT can directly and rapidly recombine with photogenerated holes from IS. The mixed crystalline phase effect of PT provides additional photogenerated electrons, consuming more photogenerated holes from IS. This not only reduces the recombination rate of photogenerated carriers from IS, but also facilitates the migration of more electrons from the higher conduction band position of IS (compared with PT) to 304SS. Consequently, the PT/IS photoelectrode shows the best PCP performance, owing to the synergistic effect between the mixed crystalline phase of rutile–anatase and the Z-scheme heterojunction structure of TiO_2_ and In_2_S_3_.

## 3. Experimental

### 3.1. Synthesis of In_2_S_3_@TiO_2_/In_2_S_3_ Heterojunction

All reagents were of analytical grade and used without additional purification. In_2_S_3_@TiO_2_/In_2_S_3_ films were synthesized on FTO glass (1.5 × 3 cm^2^, 14 Ω/cm^2^, Wuhan Lattice Solar Technology Co., Ltd., Wuhan, China) through a hydrothermal method. Specifically, the precursor solution was prepared by dissolving 6 mM indium nitrate hydrate (>99.9%, In(NO_3_)_3_∙xH_2_O, Aladdin, Shanghai, China) and 12 mM thiourea (>99%, CH_4_N_2_S, Aladdin, Shanghai, China). Next, 0.03 g of titanium dioxide nanoparticles (>99.5%, P25, Degussa, Frank, Germany) was added to the precursor solution. After 30 min of ultrasound treatment in the dark, the mixture solution and the cleaned FTO substrates were placed in a Teflon-lined autoclave, with the conductive face-down of FTO against the wall of the autoclave and immersed in the solution. The autoclave was then transferred to an oven, heated to 140 °C, and maintained for 24 h. The resulting film materials that adhered to the surfaces of FTO were washed with deionized water, and subsequently dried under vacuum condition at 50 °C for 3 h. The as-prepared In_2_S_3_@TiO_2_ (P25)/In_2_S_3_ film was denoted as PT/IS.

For comparison, a pure indium sulfide film (denoted as IS) was prepared through a similar process to that of the PT/IS samples using the same precursor solution, with the exception that no titanium dioxide was added to the precursor solution. The In_2_S_3_@TiO_2_ (anatase)/In_2_S_3_ film (denoted as AT/IS) was also synthesized using a process similar to that of the PT/IS samples, but with the replacement of titanium dioxide (>99.5%, Degussa, Frank, Germany) with anatase titanium dioxide (>99.8%, Macklin, Shanghai, China) in the precursor solution. Additionally, P25 titanium dioxide film (denoted as PT) and anatase titanium dioxide film (denoted as AT) were prepared through a simple spin-coating method. In this method, 0.03 g of titanium dioxide (P25 or anatase) was fully dispersed in 25 mL of deionized water and ultrasonically dispersed. Subsequently, 20 μL of the suspension was spin-coated onto the conductive face of the FTO substrate, and dried under vacuum condition at 50 °C for 3 h.

### 3.2. Characterization

The X-ray diffraction (XRD) patterns were obtained using a Rigaku SmartLab X-ray diffractometer (Cu Kα radiation) with a scan rate of 6°/min and the 2θ range was from 10 to 80°. The field-emission scanning electron microscope (FE-SEM, GeminiSEM 300, ZEISS, Oberkochen, Germany) and transmission electron microscope (TEM, Tecnai G2 F20 FEI, Oberkochen, Germany) were used to observe the morphology of as-obtained samples. Atomic force microscopy (AFM, Dimension Icon Bruker, Germany) was applied to test the surface roughness of samples. The optical absorption properties within the wavelength range from 300 to 800 nm were determined by using a Shimadzu spectrophotometer equipped with an integral sphere to record diffuse UV–vis absorption spectra of the samples. The X-ray photoelectron spectroscopy (XPS, PHI Quantera II, ULVAC–PHI, Kanagawa, Japan) with an Al Kα X-ray excitation source was carried out to obtain information on the elemental compositions and surface valence states of the samples, in which all the XPS spectra were calibrated according to the C1s binging energy at 284.8 eV. The photoluminescence (PL) spectra were performed on an Edinburgh Instrument FLS 920P fluorescence spectrophotometer (Edinburgh Instruments, Livingston, UK).

### 3.3. Photoelectrochemical and Photocathodic Protection Measurements

The photoelectrochemical (PEC) and photocathodic protection (PCP) experiments were performed on a CHI660E electrochemical workstation in a typical three-electrode configuration(CHI, Shanghai, China). The prepared semiconductor photoelectrode on FTO substrate served as the working electrode. The available photoelectrode area immersed in the aqueous electrolyte was controlled at 1 cm^2^. A platinum plate (1 × 1 cm^2^) and an Ag/AgCl electrode were employed as the counter electrode and reference electrode, respectively. The light source used a 300W Xe lamp with an AM 1.5 filter in order to simulate the solar light irradiation, the light intensity was 100 mW/cm^2^. The linear sweep voltammetry (LSV) measurements were performed using a voltage range of −0.6 V to 0.6 V vs. Ag/AgCl with a scanning rate of 10 mV/s under light irradiation. The electrochemical impedance spectroscopy (EIS) measurements were recorded with an amplitude of 10 mV over a frequency range of 10^−2^ to 10^5^ Hz under light irradiation, where the photoelectrodes were prepared on the surface of FTO substrate, and the electrolyte was 0.15 M Na_2_S + 0.1 M Na_2_SO_3_ solution. In addition, the PCP performance of the prepared films on FTO substrates was evaluated by measuring the OCP curves and I-t curves of the protected 304SS coupled with the photoelectrode. The corresponding experimental test devices are shown in Figure S3, in which the electrolyte of corrosion cell is a 3.5 wt% NaCl solution while the electrolyte in the photoelectrochemical cell is a 0.15 M Na_2_S + 0.1 M Na_2_SO_3_ solution.

## 4. Conclusions

In summary, we have successfully synthesized an In_2_S_3_@TiO_2_/In_2_S_3_ direct Z-scheme heterojunction photoelectrode through a simple one-step hydrothermal method. The obtained photoelectrodes were characterized by XRD, SEM, TEM, XPS, and UV DRS. The In_2_S_3_@TiO_2_/In_2_S_3_ photoelectrode with a mixed crystalline phase of rutile–anatase exhibited good photoelectrochemical cathodic performance, including a photocurrent response of 68 μA/cm^2^ and a potential drop of 372 mV when coupled with 304SS. The significantly enhanced PCP performance can be attributed to the synergistic effects between the mixed crystalline phase of rutile–anatase and the Z-scheme heterojunction structure of TiO_2_ and In_2_S_3_. The mixed crystalline phase provides a greater number of photogenerated electrons, while the Z-scheme heterojunction and the built-in electric field facilitate more effective transfer and separation of photoelectron–hole pairs. This allows the photogenerated electrons from IS to be transported directly and rapidly to 304SS. This work provides valuable insights into the positive impact of the mixed crystalline phase of TiO_2_ in Z-scheme photoelectrode materials systems and offers a reference for the potential application of photocathodic protection for steel.

## Figures and Tables

**Table 1:** The equivalent circuit model EIS results of different samples.

Samples	R_s_ (Ω)	CPE_1_, Y_0_ (S·sec^n^)	n_1_ (0 < n ≤ 1)	R_f_ (Ω)	CPE_2_, Y_0_ (S·sec^n^)	n_2_ (0 < n ≤1)	R_ct_ (kΩ)
**IS**	1.50	1.81 × 10^−7^	1.00	19.5	0.78 × 10^−5^	0.96	8.10
**PT(1)/IS**	1.51	2.08 × 10^−7^	1.00	18.0	1.22 × 10^−5^	0.96	6.95
**PT(3)/IS**	1.96	3.56 × 10^−7^	0.94	23.5	3.24 × 10^−5^	0.92	2.92
**PT(5)/IS**	1.84	3.00 × 10^−7^	0.97	20.3	2.03 × 10^−5^	0.94	4.17
**PT**	2.68	1.85 × 10^−7^	0.99	18.2	1.03 × 10^−5^	0.97	9.03

## Data Availability

Not applicable.

## Supplementary Materials

The following supporting information can be downloaded at: https://www.mdpi.com/article/10.3390/molecules28186554/s1, Figure S1: AFM images of AT/IS and PT/IS films; Table S1: The surface area roughness (R_a_) and the root-mean-square roughness (R_q_) of AT/IS and PT/IS; Figure S2: LSV curves (a) and the charge injection efficiency (b) of PT, IS and PT(3)/IS photoelectrodes under simulated sun light irradiation; Figure S3: The experimental test devices: (a) OCP, (b) I-t.
