# Supplementary material for: In2S3@TiO2/In2S3 Z-Scheme Heterojunction with Synergistic Effect for Enhanced Photocathodic Protection of Steel
Source: Molecules. 2023 Sep 10;28(18):6554. doi: 10.3390/molecules28186554 (PMC10536402; doi:10.3390/molecules28186554)
Supplement: Supplementary file 1 [file molecules-28-06554-s001.zip › molecules-2576131-supplementary.pdf]

Supplementary Materials

In_2S_3@TiO_2/In_2S_3 Z-scheme Heterojunction with Synergistic Effect for Enhanced Photocathodic Protection of 304 Stainless Steel

Authored by

Yue Chang [1,2,3,*], Kaili Suo [1], Yuhang Wang [1], Xiaona Ren [4] and Jiangli Cao [1,*]

[1] Institute for Advanced Materials and Technology, University of Science and Technology Beijing, Beijing 100083, China
[2] National Materials Corrosion and Protection Data Center, University of Science and Technology Beijing, Beijing 100083, China
[3] BRI Southeast Asia Network for Corrosion and Protection (MOE), Shunde Innovation School, University of Science and Technology Beijing, Foshan 528399, China
[4] Institute of Powder Metallurgy and Advanced Ceramics, School of Materials and Engineering, University of Science and Technology Beijing, Beijing 100083, China
* Correspondence: changyue@ustb.edu.cn (Y.C.); jlcao@mater.ustb.edu.cn (J.C.)

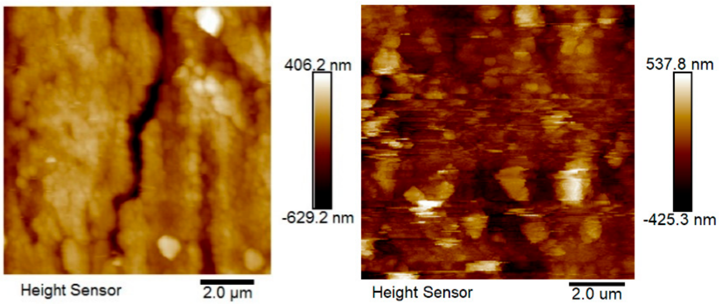

Figure S1. AFM images of AT/IS and PT/IS films.

Table S1. The surface area roughness (R_a) and the root-mean-square roughness (R_q) of AT/IS and PT/IS.

**Samples**	R_q (nm)	R_a (nm)
**AT/IS**	120	85.4
**PT/IS**	128	96

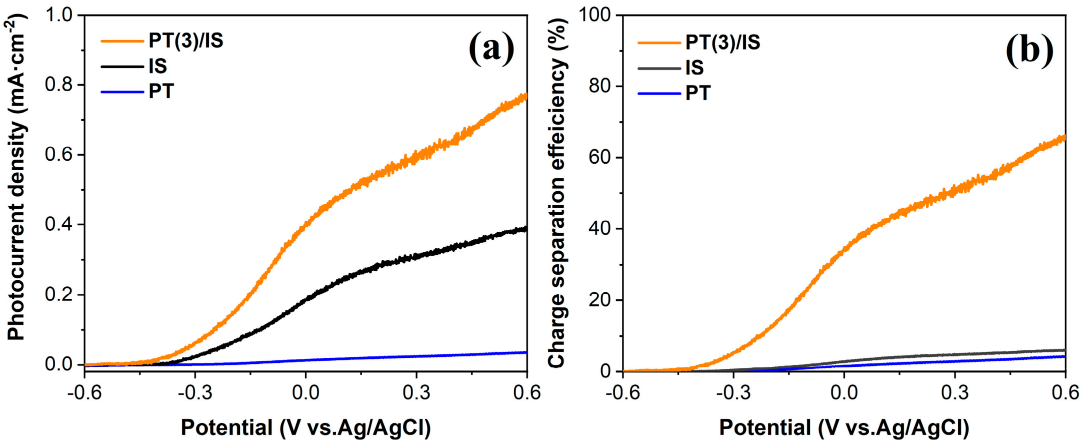

Figure S2. LSV curves (a) and the charge injection efficiency (b) of PT, IS and PT(3)/IS photoelectrodes under light irradiation.

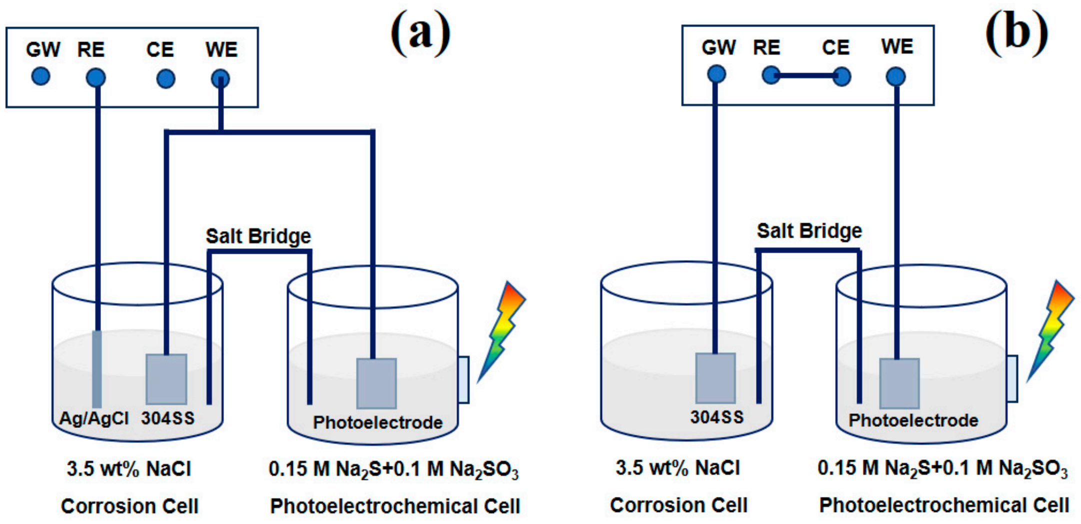

Figure S3. The experimental test devices: (a) OCP, (b) I-t
